# Hypertension prevalence and its trend in Bangladesh: evidence from a systematic review and meta-analysis

**DOI:** 10.1186/s40885-020-00143-1

**Published:** 2020-06-01

**Authors:** Mohammad Ziaul Islam Chowdhury, Meshbahur Rahman, Tanjila Akter, Tania Akhter, Arifa Ahmed, Minhajul Arifin Shovon, Zaki Farhana, Nashit Chowdhury, Tanvir C. Turin

**Affiliations:** 1 Department of Community Health Sciences, University of Calgary, TRW Building (3rd Floor), 3280 Hospital Drive NW, Calgary, Alberta T2N 4Z6 Canada; 2 Department of Statistics, Shahjalal University of Science and Technology, Sylhet, Bangladesh; 3 Department of Family Medicine, University of Calgary, Calgary, Alberta Canada

**Keywords:** Hypertension, Prevalence, Trend, Bangladesh

## Abstract

**Background:**

Hypertension, itself being a major chronic condition, is one of the most significant risk factors for premature cardiovascular diseases and mortality. Hypertension is responsible for 13% of global deaths and three-quarters of the world’s hypertensive population reside in low- and middle-income countries. Bangladesh is one of those countries that experiencing an epidemiological transition from communicable to non-communicable diseases, a nutritional transition from a traditional diet to process and fast food, and an increase in a sedentary lifestyle, resulting in increased hypertension prevalence. We carried out a systematic review and meta-analysis to identify existing research on hypertension prevalence in Bangladesh, summarize findings and assess its temporal change.

**Methods:**

We searched MEDLINE, EMBASE and PubMed and relevant references to identify studies on the prevalence of hypertension in Bangladesh. We used Random-effects meta-analysis to pool the prevalence estimates and performed subgroup analyses. We assessed heterogeneity, a trend in prevalence of hypertension and publication bias in selected studies.

**Results:**

Our search initially identified 735 articles and after removing duplicates, reviewing titles and abstracts, and screening full texts, 53 studies were finally selected. The studies comprised 305,432 subjects and reported overall, gender-specific, geographical location specific and criteria specific prevalence of hypertension. We identified the range of hypertension prevalence is from 1.10% to 75.0% and the overall weighted pooled prevalence of hypertension is 20.0%. An extremely high heterogeneity (I^2^ = 99.53%; Cochran Q-statistic p < 0.001) was observed in the prevalence of hypertension. Consequently, we performed subgroup analysis based on gender, age group and geographical location of the study participants, the cut-off level used to define hypertension, and the types of hypertension reported and presented our findings accordingly. An overall increasing trend of hypertension prevalence is also observed.

**Conclusions:**

The prevalence of hypertension is high and rising in Bangladesh. Strategies targeting prevention are required to mitigate a further increase in the prevalence and reduce the morbidity and mortality associated with it.

## Introduction

Hypertension, itself being a major chronic condition, is one of the most significant risk factors for premature cardiovascular diseases and mortality [1]. Defined as a persistent elevation of blood pressure (BP) beyond 130/80 mmHg [2], hypertension is directly related to the development of several fatal conditions such as stroke, coronary artery disease, heart failure, atrial fibrillation, and peripheral vascular disease [3]. Persistent untreated hypertension can also lead to kidney failure, dementia and cognitive decline [4, 5]. Moreover, increased blood pressure can be considered as a marker for other risk factors of non-communicable diseases (NCD) such as increasing body weight, dyslipidemia, glucose intolerance, and the metabolic syndrome [6]. Hypertension has been attributed to be responsible for 13% of global deaths [7]. With the projection of a 30% increase in worldwide prevalence of this condition by the year 2025 and for its pivotal role in the rising global burden of disease and disability, hypertension has become one of the most challenging concerns for world public health [8–10].

Contrary to the popular belief that NCD such as hypertension afflict mostly high-income countries, nearly 80% of the NCD deaths occur in low- and middle-income countries [11]. Studies estimate that three-quarters of the world’s hypertensive population reside in low- and middle-income countries [10] and the prevalence of hypertension is higher in low- and middle-income countries (31.5%) than in high-income countries (28.5%) [10]. Bangladesh is one of those low- and middle-income countries that experiencing an epidemiological transition from communicable to non-communicable diseases [12]. On top of that, the ongoing nutritional transition from a traditional diet to process and fast food, increasing trends of sedentary lifestyle due to improved socio-economic status, congested living conditions and absence of physical movement due to rapid unplanned urbanization may contribute largely to the emergence of hypertension epidemic in Bangladesh. Although Bangladesh successfully combatted against major communicable diseases, this altered pattern of diseases throws a paramount challenge to the health care system of Bangladesh.

Bangladesh currently lacks a population-based surveillance system to track the burden of any non-communicable chronic disease including hypertension [13, 14]. In addition, the absence of nationwide population-based surveys or central administrative health data restricts accurate information on the prevalence of diseases like hypertension [13, 14]. Although a handful of studies on the prevalence of hypertension among the Bangladeshi population was conducted, their findings are not consistent. Further, there is also shortage of research that synthesizes existing literature on hypertension prevalence through proper systematic review and meta-analysis. Prevalence estimation in general is based on single large-scale representative survey. Considering absence of accessible nationally representative large-scale surveys in Bangladesh, we choose alternative approach to synthesize existing information on hypertension prevalence from the scientific literature through meta-analysis. The purpose of our study is to summarize existing knowledge through systematic review and provide combined/summary prevalence estimates (a combined estimate derived from multiple similar studies that presented same effect measure) using meta-analysis. Meta-analysis, a method to obtain a weighted average of results from various studies offer several advantages. It can provide a more stable estimate than single independent studies because of the increased amount of data uses and hence provides more statistical power to detect effects. Further, it also helps researchers identify inconsistencies/heterogeneity in research findings and explore factors that may explain the sources for these discrepancies. Inadequate information on the magnitude of the condition restrains the health professionals and policymakers from substantiating its extent and undertaking of management plan afterward. To illustrate the actual picture of the current situation, lay bare the discrepancies among hypertension prevalence reported by different individual studies, and make the readers aware of this fact, we carried out this systematic review and meta-analysis using data already published in the literature. Our objective is to assess the prevalence of hypertension in Bangladesh, its trend and to provide comprehensive information that can be used for planning and executing successful preventive strategies for this condition.

## Methods

### Data sources and search strategy

We systematically searched MEDLINE, EMBASE, and PubMed from inception to May 13, 2019, for studies on the prevalence of hypertension among the Bangladeshi adult population. We also searched the reference lists of all relevant publications for information about other potential studies. We limited inclusion to studies published in English. The search strategy focused on three key elements: hypertension, prevalence, and Bangladesh. The search strategy is provided in detail in Table 1.

**Table 1:** Search strategy used in different databases

MEDLINE	PubMed	EMBASE
1. exp Hypertension/ 2. high blood pressure.mp. 3. exp Blood Pressure/ 4. hyperten*.mp 5. 1 or 2 or 3 or 4 6. exp Prevalence/ 7. exp Bangladesh/ 8. 5 and 6 and 7	((Bangladesh) AND Prevalence) AND ((((Hypertension) OR High blood pressure) OR Blood pressure) OR Hyperten*)	1. exp hypertension/ 2. high blood pressure.mp. 3. exp blood pressure/ 4. hyperten*.mp. 5. 1 or 2 or 3 or 4 6. exp prevalence/ 7. exp Bangladesh/ 8. 5 and 6 and 7

### Study selection

Two reviewers independently using a two-step process identified potentially eligible articles. At first, titles and abstracts were screened. Abstracts were retained if they reported an original study on the prevalence of hypertension in Bangladesh. Review articles were not considered. At this stage, an abstract was retained either when the reviewers agreed that it should be or when there was uncertainty on eligibility based on title and abstract alone. Selected abstracts were subsequently screened based on a full-text review. A broad inclusion criterion was used to provide a comprehensive systematic review of the topic. No restrictions were imposed on study type (e.g., cohort study, cross-sectional study), geographical region (e.g., urban, rural), time period or age groups. There was also no restriction on diagnostic criteria used to define hypertension. A study was included if the prevalence of hypertension was reported in the general adult Bangladeshi population but excluded if the prevalence was reported on individuals with specific diseases (e.g., diabetes). Studies were excluded that did not report the prevalence of hypertension, duplicate, non-human studies, and studies involving children. Agreement between reviewers was quantified. Any disagreement between reviewers was resolved through consensus.

### Data extraction and data items

The following information was extracted from the included studies: author and year of publication; age range of the participants; gender and number of participants; area (urban/rural) in which the study was carried out; sample selection procedure; study design; method for diagnosis of hypertension; cut-off level used to define hypertension and the prevalence of hypertension. Two reviewers independently extracted data using a predefined standardized form.

### Summary measures

The summary statistics from the individual studies were the prevalence of hypertension defined as the number of people in the sample with hypertension, divided by the total number of people in the sample. The prevalence of hypertension could differ considerably depending on the definition of hypertension. As such, we consider all the definitions of hypertension and report the summary results (pooled prevalence) separately based on the definition used to define hypertension. We also presented separate summary results according to the prevalence type, gender, age and geographical location of the study participants assuming potential substantial prevalence differences within these categories.

### Statistical analysis

The pooled prevalence estimates for hypertension and all subgroup analyses and their 95% confidence intervals were calculated using a random-effects model according to the cut-off level used to define hypertension, types of prevalence reported, gender, age group and geographical location of the study participants. A random-effects meta-analysis model assumes the observed estimates of prevalence (treatment effect) can vary across studies because of both the real differences (heterogeneity) and sampling variability (chance) in the prevalence in each study [15]. Heterogeneity and consistency were assessed using Cochran’s Q test and the I^2^ statistic. Cochran’s Q is the classical measure of heterogeneity, which is calculated as the weighted sum of squared differences between individual study prevalence and the pooled prevalence across studies, with the weights being those used in the pooling method [16, 17]. I^2^ measures the percentage of variability in prevalence estimates that is due to between-study heterogeneity rather than chance [16, 17]. Small study effects were examined using a funnel plot and Egger’s test. Inter-rater reliability was measured. All statistical analyses were performed using Stata version 13.1 (Stata Corp, College Station, TX) using the metaprop, metareg, metabias, and metafunnel commands.

## Results

### Study selection

**Fig. 1:**
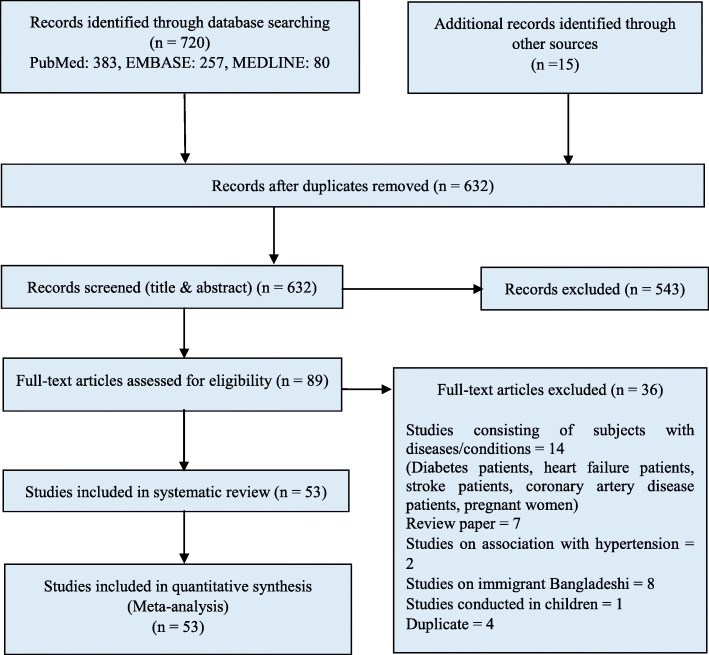
PRISMA diagram for the systematic review of studies that evaluated the prevalence of hypertension in Bangladesh

We identified 720 studies on the prevalence of hypertension in Bangladesh through electronic search and a further 15 potentially relevant studies through a grey literature search. After removing duplicates and reviewing titles and abstracts, 89 articles remained for full-text screening. The main reason for exclusion was irrelevance with our study objective. Of the 89 articles full text screened, 36 were excluded for the following reasons: 14 were conducted on subjects with diseases, 7 were review articles, 2 studies assessed the association with hypertension, 8 studies conducted on Bangladeshi immigrants living abroad, 1 was carried out on children and 4 were duplicate studies. Consequently, 53 studies were finally selected for this systematic review. There was good agreement (84.21%) between reviewers on the primary articles eligible for inclusion. The article selection process is shown in Fig. 1.

### Study characteristics

A summary describing the characteristics of the selected studies on the prevalence of hypertension in Bangladesh is presented in Table 2. Twenty-one studies were conducted in rural populations, 8 studies were conducted on urban populations, and 24 studies in both rural and urban populations. Thirteen different cut-off levels or criterion was used to define hypertension and “≥ 140/90 mmHg and/or anti-hypertensive medication” was the most common cut-off value with 22 studies reported this cut-off level. Both genders were represented in almost all studies except 4 studies where study participants were female only. Age of the study participants varied with “≥ 35 years” reported by the highest number of studies (13 studies) followed by “≥ 25 years” (11 studies) and “≥ 18 years” (8 studies) respectively. The study design was cross-sectional in most studies (20 studies) and the cluster-sampling technique was the most commonly used sample selection procedure.

**Table 2:** Characteristics of studies that evaluated the prevalence of hypertension in Bangladesh

Study /Author	Year Published	Age Range	Gender	Sample Size	Study Area (Urban/Rural)	Study Design /Sampling Method	Hypertension Diagnosis Method	SBP/DBP Cut-off Value (mmHg) for Hypertension	Prevalence (%) with 95% CI
Zaman et al. [18]	2001	≥ 18 years	Both	510	Rural	Random Sampling	Mean of two measurements at 2-minute intervals by physicians	≥ 140/90 and ≥ 160/95	At Cut-off ≥ 140/90 mmHg: 9.8% (Men), 15.6 % (Women), 12.9% (Total); At Cut-off ≥ 160/95 mmHg: 4.9% (Men), 6.5% (Women), 5.8% (Total)
Zaman et al. [19]	2016	≥ 25 years	Both	9,275	Both urban and rural	Random Sampling with probability proportionate to size	Mean of two measurements at 2-minute intervals	≥ 140/90 and/or anti-hypertensive medication	Unadjusted: 18.5% (Men), 17.3% (Women), 17.9% (Total); Age adjusted: 19.1% (Men), 23.0% (Women), 20.8% (Total)
Zaman et al. [20]	2015	≥ 25 years	Both	4,073	Both urban and rural	Multistage Cluster Sampling	Mean of two measurements at 2-minute intervals	≥ 140/90	Unadjusted: 20.6% (Men), 22.1% (Women), 21.4% (Total); Adjusted: 20.7% (Men), 25.0% (Women), 23.1% (Total)
Minh et al. [21]	2009	25 - 64 years	Both	2,061 (Matlab), 2,028 (Mirsarai), 1,983 (Abhoynagar); 1,997 (WATCH)	Rural	A multi-site Cross-sectional Study	Three measurements with average of the last two readings	≥ 140/90 or treatment with BP medication; ≥ 160/100 or treatment with BP medication	Matlab at Cut-off ≥ 140/90 mmHg: 12.5% (Men), 21.0% (Women), 17.1% (Total); Matlab at Cut-off ≥ 160/100 mmHg: 5.0% (Men), 12.4% (Women), 9.1% (Total); Mirsarai at Cut-off ≥ 140/90 mmHg: 20.3% (Men), 27.4% (Women), 24.1% (Total); Mirsarai at Cut-off ≥ 160/100 mmHg: 12.3% (Men), 18.0% (Women), 15.3% (Total); Abhoynagar at Cut-off ≥ 140/90 mmHg: 13.3% (Men), 19.8% (Women), 16.8% (Total); Abhoynagar at Cut-off ≥ 160/100 mmHg: 7.5% (Men), 12.6% (Women), 10.2% (Total); WATCH at Cut-off ≥ 140/90 mmHg: 7.4% (Men), 11.2% (Women), 9.3% (Total); WATCH at Cut-off ≥ 160/100 mmHg: 2.6% (Men), 6.3% (Women), 4.5% (Total);
Tareque et al [22]	2015	≥ 35 years	Both	7,499	Both urban and rural	Two stage Stratified Sampling	Three measurements with average of the second and third measurements	≥ 140/90 and/or anti-hypertensive medication	20.0% (Men), 32.0% (Women), 27.1% (Total), 23.8% (Rural), 32.7% (Urban)
Sayeed et al. [23]	2003	≥ 20 years	Both	5,055	Rural	Purposive Cluster Sampling	Measured by a single investigator using mercury sphygmomanometer	SBP ≥ 140	NR
Sayeed et al. [24]	2002	≥ 20 years	Both	2,361	Both urban and rural	Cross-sectional Study	Mean of two measurements at 2-minute intervals	≥ 140/90	14.4% (Systolic hypertension), 9.1% (Diastolic hypertension)
Razzaque et al. [25]	2011	25 - 64 years	Both	2,000	Rural	WHO STEPS Methodology	Three measurements of 5 minutes interval with average of the last two readings	High Blood Pressure: ≥ 140/90; Raised Blood Pressure: ≥ 140/90; or using anti-hypertensive medication; Self-Reported: Informed by physician	High Blood Pressure: 10.6% (Male), 13.9% (Female); Raised Blood Pressure: 12.5% (Male), 21.0% (Female); Self-Reported: 6.8% (Male), 14.8% (Female)
Rahman et al. 2015 [26]	2015	≥ 35 years	Both	7,876	Both urban and rural	Multistage Stratified Cluster Sampling	NR	≥ 140/90 and/or anti-hypertensive medication	19.1% [17.6–20.7] (Men), 31.7% [29.9–33.5] (Women), 24.4% (Overall Age-standardized), 25.4% (Crude)
Quasem et al. [27]	2001	≥ 60 years	Both	240	Both urban and rural	Random Multistage Cluster Sampling	Average of two readings	≥ 140/90 and/or anti-hypertensive medication	75% [69 – 80] (Urban Site), 53% [47 – 59] (Rural Site)
Parr et al. [28]	2011	≥ 25 years	Both	8,591	Both urban and rural	Cross-Sectional Study	NR	NR	12.4% (Rural), 16.1% (Urban)
Alam et al. [29]	2014	≥ 20 years	Both	1,678	Both urban and rural	Random selection	Three measurements at 5 minutes interval with average of the second and third measurements	SBP ≥ 140 or DBP ≥ 90 or both	17.1% (Total), 23.6% (Urban), 10.8% (Rural)
Huda et al. [30]	2012	15 - 65 years	Both	1,000	Urban	Multistage Clustered Sampling	NR	≥ 140/90 or anti-hypertensive medication	11.60%
Khanam et al. [31]	2015	≥ 25 years	Both	6,094	Rural	Representative Sample of a Cross-sectional Study	Three measurements at 5 minutes interval with the averages ofthe last two measurements	≥ 140/90 or anti-hypertensive medication	16% (Total), 13.5% (Men), 18.4% (Women)
Khanam et al. [32]	2015	≥ 25 years	Both	6,082	Rural	Cross-sectional Study	Three measurements with the averages of the last two readings	≥ 140/90 or anti-hypertensive medication	14.9% (Self-reported), 11.1% (Undiagnosed), 9.7% (Men undiagnosed), 12.6% (Women undiagnosed)
Khanam et al. [33]	2014	≥ 25 years	Both	29,960	Rural	Cross sectional Door to Door Survey	Not based on blood pressure measurements	Face-to face interviews with a structured questionnaire	13.7% (Total), 8.9% (Male), 14.8% (Female)
Jesmin et al. [34]	2013	≥ 15 years	Female	1,802	Rural	Cross-sectional Study/Stratified Multistage Random Sampling	Average of two measurements at 15 minute interval	≥ 130/85 or anti-hypertensive medication	28.2% (Overall)
Jesmin et al. [35]	2012	≥ 15 years	Female	1,535	Rural	Cross-sectional Study/Stratified Multistage Random Sampling	Average of two measurements at 15 minute interval	≥ 130/ 85	29.10%
Islam et al. [36]	2015	≥ 25 years	Both	730	Urban	Cross-sectional Study/Multi-stage Random Sampling	Three measurements at 5 minutes interval with average of the second and third measurements	≥ 140/90 or self-reported or anti-hypertensive medication	23.7% (Overall age-adjusted), 23.6% (Male age-adjusted), 21.7% (Female age-adjusted)
Islam et al. [37]	2012	≥ 30 years	Both	1,004	Rural	A cross-sectional Study/ Purposive Selection (area), Random Selection (households)	Lowest value of the three measurements.	≥ 140/90	6.6% [5.1 – 8.3] (Overall), 8.2% (Male), 5.8% (Female)
Islam [38]	2016	≥ 35 years	Both	7,561	Both urban and rural	Stratified Multistage Cluster Sampling	Three measurements at 10 minutes interval with the averages of the last two measurements	≥ 140/90 and/or anti-hypertensive medication	11.9%
Islam et al. [39]	2015	≥ 30 years	Both	3,096	Rural	Multilevel Cluster Random Sampling	Average of the two readings at 5 minute interval	≥ 140/90 or self-report of using medication	40% [38–42] (Crude), 40.3% (Male Total), 40% (Female Total)
Hasan et al. [40]	2012	18 - 65 years	Both	1,240	Rural	Cross-sectional Study/Three stage Sampling; Purposively (first stage) and Simple Random Sampling (second and third stage)	Two measurements	≥ 140/90 or self-report of using medication	19.3%
Karim et al. [41]	2013	18 - 65 years	Both	1,134	Rural	Cross-sectional study/ Direct Interview Using Structured Questionnaire	Average of the two readings at two minutes interval	≥ 140/90	19.1%
Harshfield et al. [42]	2015	≥ 35 years	Both	8,834	Both urban and rural	Two Stage Sampling: Probability Proportional to the Size (first stage), Systematic Sampling (second stage)	Average of the three measurements	≥ 140/90 (Measurement Only); ≥ 140/90 or diagnosed by health professional or blood pressure lowering medication (Medical)	Measurement Only: 19.1% (Overall), 14.5% (Male), 24.1% (Female); Medical: 26.4% (Overall), 19.6% (Male), 33.6% (Female)
Fatema et al. [43]	2016	31 - 74 years	Both	62,538 (Not high risk for CVD), 1,170 (High risk for CVD)	Rural	Purposive Sub-cohort from a Cohort Study	Single reading	≥ 140/90 or receiving treatment for hypertension	9.0% (Not high risk for CVD participants), 4.4% (High risk for CVD participants)
Fatema et al. [44]	2015	31-74 years	Both	1,170 (High risk for CVD), 563 (Not high risk for CVD)	Rural	Cohort Study/Participants who agreed to take part and random selection	Average of the three readings at 5 minutes interval	≥ 140/90	15.8% (High risk group), 3.6% (Not high risk group)
Chowdhury et al. [45]	2016	≥ 35 years	Both	7,839	Both urban and rural	Cross-sectional Study/Two-stage Stratified Cluster Sampling	Three measurements of 10 minutes interval with average of the second and third measurements	≥ 140/90 or anti-hypertensive medication	26.4% (Overall), 20.3% (Men), 32.4% (Women)
Hasan et al. [46]	2012	18-65 years	Both	1,240	Rural	Three Stage Sampling	NR	NR	19.3%
Chakrabarti et al. [47]	2015	≥ 30 years	Both	3,104	Rural	Cluster Random Sampling	Average of two measurements at 5-minute intervals	≥ 140/90	7.4%
Bishwajit et al. [48]	2016	≥ 35 years	Female	2,022	Both rural and urban	Two Stage Sampling: Probability Proportional to the Size (first stage), Systematic Sampling (second stage)	Three measurements of 10 minutes interval with average of the second and third measurements	≥ 140/90	18%
Bhowmik et al. [49]	2012	≥ 20 years	Both	2,293	Rural	Cross-sectional Study/Random Selection	Mean of the two measurements at 5-minutes intervals	≥ 140/90 or anti-hypertensive medication	15.5% (Total), 17.5% (Male), 14.3% (Female)
Alam et al. [50]	2014	≥ 20 years	Female	1,600	Both rural and urban	Longitudinal Study/ Random Selection	Three measurements of 5 minutes interval with average of the second and third measurements	≥ 140/90 or anti-hypertensive medication	17.1% (Overall), 23.6% (Urban), 10.8% (Rural)
Moni et al. [51]	2010	≥ 60 years	Both	317	Urban	Cross-sectional Study/Convenient Sampling Technique	NR	≥ 140/90 or anti-hypertensive medication	44.8% (Overall), 53.5% (Male), 38.8% (Female)
Das et al. [52]	2010	16-65 years	Both	1,200	Urban	Cross-sectional Study/Multi-stage Cluster Sampling	NR	> 139/89	17.3%
Sayeed et al. [53]	1994	≥ 15 years	Both	1,005	Rural	Cluster Sampling	Mean of the three measurements	≥ 140/90	10.5% (SBP), 9.0% (DBP)
Islam et al. [54]	1983	≥ 10 years	Both	5,026	Rural	NR	Single measurement in sitting position	DBP ≥ 90	6.7% (DBP)
Islam et al. [55]	1979	18-55 years	Both	8,172	Urban	Total of a specific population	Single measurement in sitting position	DBP ≥ 90	13.3% (DBP)
Malik A [56].	1976	All age	Both	7,062	Both rural and urban	NR	NR	NR	1.10%
Mondal et al. [57]	2013	≥ 18 years	Both	481	Both rural and urban	Cross -sectional study/Random Selection	Three measurements at 10 minutes interval with average of the last two measurements	≥ 140/90	33.3%
Sayeed et al. [58]	1995	≥ 15 years	Both	1,005	Rural	Cluster Sampling	Mean of the three measurements	SBP ≥ 140, DBP > 90	10.5% (SBP), 9.0% (DBP)
Khalequzzaman et al. [59]	2017	18 - 64 years	Both	2,551	Urban	Stratified Simple Random Sampling	Three measurements with the mean of the second and third readings	≥ 140/90 or use of anti-hypertensive medication	18.6% [16.5 - 20.8] (Men), 20.7% [18.5 - 22.9] (Women)
Ali et al. [60]	2018	21 - 23 years	Both	184	Urban	Cross-sectional Study	Mean of the two measurements at 5 minutes interval	≥ 140/90	6.5% (Overall), 12.1% (Male), 3.4% (Female)
Biswas et al. [61]	2019	≥ 35 years	Both	8,763	Both rural and urban	Cross-sectional Study/Two-stage Cluster Probability Random Sampling	NR	≥ 140/90 or anti-hypertensive medication	27% (Overall), 25.3% [23.5 - 27.1] (Rural), 33.3% [31.1 - 35.5] (Urban)
Kibria et al. [62]	2018	≥ 35 years	Both	7,839	Both rural and urban	Two Stage Stratified Sampling: Probability Proportional to the Size (first stage), Systematic Sampling (second stage)	Three measurements at 10 minutes interval with average of the last two measurements	≥ 130/80 (2017 ACC/AHA Guideline); ≥ 140/90 (the JNC7 Guideline)	The JNC7: 25.7% [24.5 – 27.0] (Overall Crude), 19.4% [18.0 - 21.0] (Male Crude), 31.9% [30.1 - 33.6] (Female Crude); 2017 ACC/AHA: 48% [46.4 – 49.7] (Overall Crude), 41.4% [39.4 - 43.5] (Male Crude), 54.5% [52.4 - 56.4] (Female Crude)
Islam et al. [63]	2018	≥ 18 years	Both	1,843	Both rural and urban	Multistage, Geographically Clustered, Probability-based Sampling	Average of two measurements at 2 minute interval	≥ 130/80 or a self-reported diagnosis of hypertension (2017 ACC/AHA Guideline); ≥ 140/90 (The JNC7 Guideline)	The JNC7: 17.9% [16.2 – 19.7] (Overall), 16.4% [14.0 - 18.9] (Male), 19.3% [16.9 - 22.0] (Female) 14.6% [12.5 - 16.7] (Rural), 23.2% [20.1 - 26.5] (Urban); 2017 ACC/AHA: 40.7% [38.5 – 43.0] (Overall), 43.4% [40.1 - 46.7] (Male), 38.3% [35.2 - 41.4] (Female), 36.1% [33.3 - 38.9] (Rural), 48.0% [44.3 - 51.8] (Urban)
Rawal et al. [64]	2017	≥ 25 years	Both	507	Urban	Cross-sectional Study/Purposive Selection	Average of two measurements at 2 minute interval	≥ 140/90	13.7% [19.31 - 26.02] (Overall), 12.6% (Male), 14.8% (Female)
Roy et al. [65]	2019	≥ 35 years	Both	7,307	Both rural and urban	Two-stage Stratified Cluster Sampling: Probability Proportional to the Size (first stage), Systematic Sampling (second stage)	Three measurements at 10 minutes interval with average of the second and third measurements	≥ 140/90 or anti-hypertensive medication	24.7% [23.6–25.8] (Overall), 18.5% [17.1–20.0] (Male), 30.8% [29.1–32.5] (Female), 22.7% [21.5–24.0] (Rural), 31.3% [29.0–33.6] (Urban)
Ahmed et al. [66]	2019	≥ 35 years	Both	8,835	Both rural and urban	Two-stage Stratified Sampling: Probability Proportional to the Size (first stage), Systematic Sampling (second stage)	Three measurements at 10 minutes interval with average of the last two measurements	≥ 140/90	19.1%
Kibria et al. [67]	2018	≥ 35 years	Both	7,839	Both rural and urban	Two-staged Cluster Sampling Design	Three measurements at 10 minutes interval with average of the second and third measurements	≥ 140/90 or anti-hypertensive medication	Combined: 25.7% (Overall); Urban: 32.6% [30.5–34.8] (Overall), 25.1% (Male), 40.2% (Female); Rural: 23.6% [22.5–24.7] (Overall), 17.6% (Male), 29.4% (Female)
Kibria et al. [68]	2018	≥ 35 years	Both	7,839	Both rural and urban	Two-staged Sampling Design	Three measurements at 10 minutes interval with average of the last two measurements	≥ 130/80 or taking blood pressure lowering drug (2017 ACC/AHA Guideline); ≥ 140/90 or taking blood pressure lowering drug (The JNC7 Guideline)	JNC7: 25.7% (Overall); 2017 ACC/AHA: 48% (Overall)
Rahman et al. [69]	2018	≥ 25 years	Both	9,275	Both rural and urban	Multistage, Geographically Clustered, Probability-based Sampling	Average of two measurements at 2 minute interval	≥ 140/90 or anti-hypertensive medication	20.1% (Overall), 20.3% (Male), 19.9% (Female), 17.9% (Rural), 22.2% (Urban)
Biswas et al. [70]	2017	≥ 35 years	Both	7,544	Both rural and urban	NR	NR	≥ 140/90 or anti-hypertensive medication	26.5%

The studies selected in this systematic review comprised 305,432 subjects. Studies reported overall prevalence, gender-specific prevalence, geographical location specific prevalence and criteria specific prevalence of hypertension that ranged from 1.10% to 75.0%. The weighted pooled prevalence of hypertension regardless of gender, age group and geographical location of the study participants, the cut-off level used to define hypertension, and the types of hypertension reported, was 20.0% [95% CI: 18% - 21%]. There was an extremely high heterogeneity observed in the prevalence of hypertension (I^2^ = 99.53%; Cochran Q-statistic p < 0.001). One approach to comprehend this problem of high heterogeneity is to identify the factors that may explain the heterogeneity, stratify studies into more homogeneous subgroups accordingly, perform subgroup analysis and finally present corresponding results. Presenting results in subgroups will help overcome the issue of heterogeneity, results will be more comparable and more informative to the readers to get an in-depth notion about hypertension prevalence in Bangladesh. This will also make readers more aware about potential variability in hypertension prevalence. We performed subgroup analysis based on gender, age group and geographical location of the study participants, the cut-off level used to define hypertension, and the types of hypertension reported and presented our findings accordingly.

### Prevalence of hypertension according to the gender of study participants

**Fig. 2:**
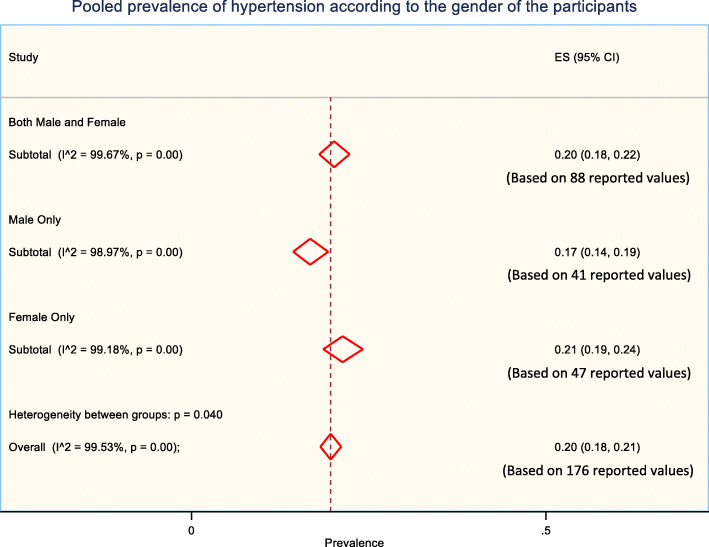
The pooled prevalence of hypertension according to the gender of the participants

The prevalence of hypertension was observed higher in females compared to males. Weighted pooled prevalence of hypertension was 17% [95% CI: 14% - 19%]) among males, 21% [95% CI: 19% - 24%]) among females and 20% [95% CI: 18% - 22%] among combined males and females (Fig. 2).

### Prevalence of hypertension according to the age group of study participants

**Fig. 3:**
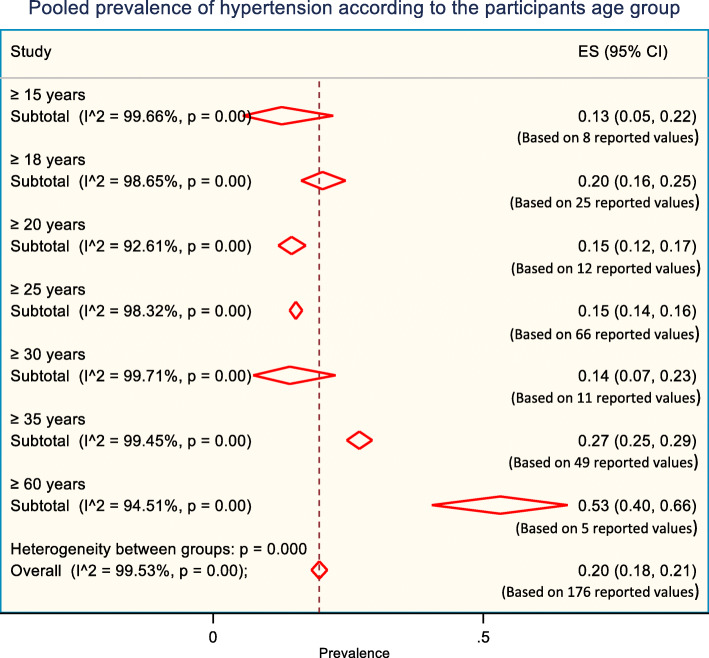
The pooled prevalence of hypertension according to the participant's age group

Generally, hypertension increases with age, which is also evident in this study. Initially, we identified hypertension prevalence reported in 14 different age groups. However, to obtain a pooled prevalence estimate, we merged a few age groups based on their similarities. For example, studies that reported participants age as “15 – 65 years” is merged with “≥ 15 years age” group or studies where participants age was reported as “18 – 65 years” is merged with “≥ 18 years age” group. Weighted pooled prevalence of hypertension was lowest 13% [95% CI: 5% - 22%]) in “≥ 15 years age” group, while highest 53% [95% CI: 40% - 66%]) in “≥ 60 years” age group (Fig. 3).

### Prevalence of hypertension according to the geographical location of study participants

**Fig. 4:**
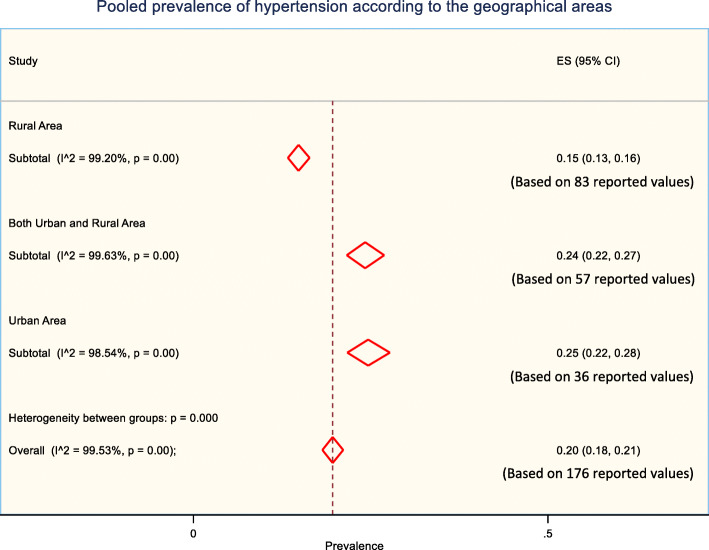
The pooled prevalence of hypertension according to the geographical areas

The prevalence of hypertension and its risk factors could vary substantially in urban and rural areas, particularly in developing countries like Bangladesh due to differences in lifestyles. Weighted pooled prevalence of hypertension was higher in urban areas (25% [95% CI: 22% - 28%]) compared to rural areas (15% [95% CI: 13% - 16%]) (Fig. 4). When study participants were from both urban and rural areas, the weighted pooled prevalence of hypertension was reported 24% [95% CI: 22% - 27%] (Fig. 4).

### Prevalence of hypertension according to the cut-off levels used to define hypertension

**Fig. 5:**
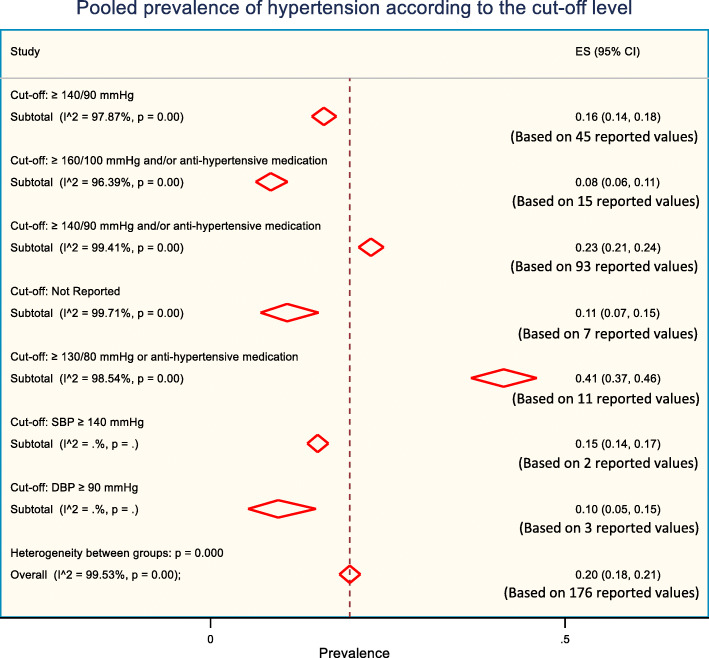
The pooled prevalence of hypertension according to the cut-off level used to define hypertension

The prevalence of hypertension varies according to the cut-off level used to define hypertension in studies. The differences in the cut-off levels for hypertension in the included studies provided a different level of hypertension prevalence (Fig. 5). Although we initially identified 13 different cut-off values/criteria used by the different studies to define hypertension, we merged them into 7 categories according to the similarities of the cut-off values. For example, cut-off value “≥ 140/90 mmHg or self-reported or anti-hypertensive medication” and “≥ 140/90 mmHg or diagnosed by health profession or anti-hypertensive medication” was merged into cut-off value “≥ 140/90 mmHg and/or anti-hypertensive medication” due to their apparent similarity. Highest prevalence was reported 41% for “≥ 130/80 mmHg and/or anti-hypertensive medication” cut-off value while lowest was reported 8% for “≥ 160/100 mmHg and/or anti-hypertensive medication” cut-off value.

### Prevalence of hypertension according to the types of prevalence

**Fig. 6:**
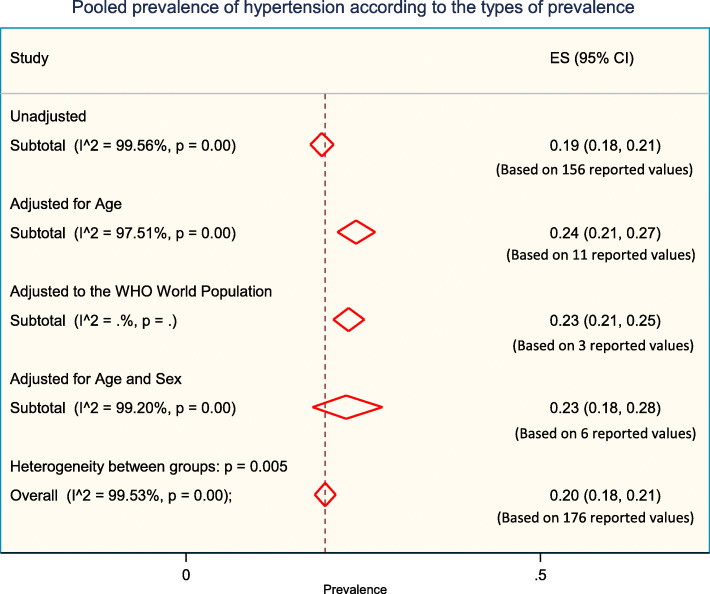
The pooled prevalence of hypertension according to the types of prevalence reported

Weighted pooled prevalence of hypertension was 19% [95% CI: 18% - 21%]) when unadjusted, 24% [95% CI: 21% - 27%]) when adjusted for age, 23% [95% CI: 21% - 25%] when adjusted to the WHO world population, and 23% [95% CI: 18% - 28%] when adjusted for age and sex (Fig. 6).

### Assessment of temporal change in hypertension prevalence

**Fig. 7:**
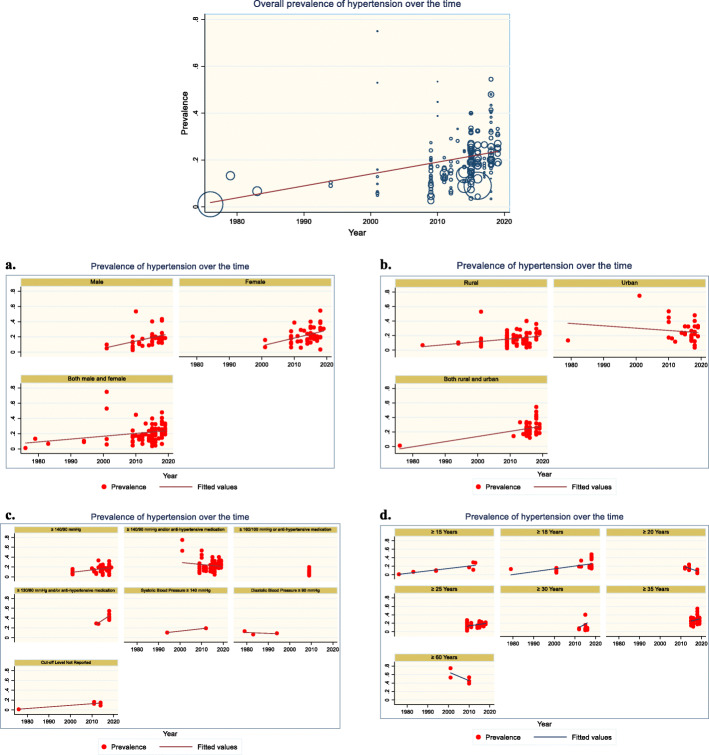
The overall prevalence of hypertension over time. **a.** Prevalence of hypertension over time according to the gender of the study participants. **b.** Prevalence of hypertension over time according to the geographical location of the study participants. **c.** Prevalence of hypertension over time according to the cut-off level used to define hypertension. **d.** Prevalence of hypertension over time according to the age group of the study participants

The time span among the studies that were conducted ranges from 1976 to 2019. However, the majority of the studies are recent, mostly conducted after 2010. To assess the temporal change in hypertension prevalence, we performed meta-regression on hypertension prevalence over the study year. The overall prevalence of hypertension increased by 0.51% (p < 0.001; Fig. 7) for every 1-year increase in the study year, suggesting that the prevalence of hypertension is on the rise. However, this increasing trend was not uniform in all groups and categories and there are instances where a decreasing trend was observed. The increase in hypertension prevalence over the time was observed quite similar both in males and in females (Fig. 7a). Over time, an increase in hypertension prevalence was observed in rural areas while a decrease was observed in urban areas (Fig. 7b). An increasing trend in hypertension prevalence was observed when cut-off levels to define hypertension were “≥ 140/90 mmHg”, “≥ 130/80 mmHg and/or anti-hypertensive medication”, “systolic blood pressure ≥ 140 mmHg”, and “unspecified” while a decreasing trend was observed in cut-off levels “≥ 140/90 mmHg and/or anti-hypertensive medication” and “diastolic blood pressure ≥ 90 mmHg” (Fig. 7c). In addition, an increasing trend in hypertension prevalence was observed in all age group categories except “≥ 20 years” and “≥ 60 years” age group categories where a decline was observed (Fig. 7d).

### Publication bias

**Fig. 8:**
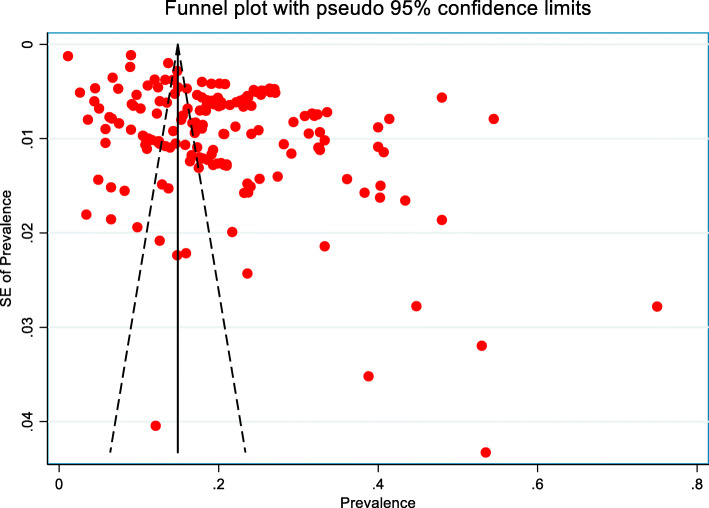
Funnel plot for the publication bias of the studies that evaluated the prevalence of hypertension in Bangladesh

The funnel plot indicated the existence of asymmetry and publication bias (Fig. 8) and Egger’s test (p < 0.001) suggested the presence of small-study effects, in which studies of smaller cohorts reported a higher prevalence of hypertension. Funnel plot asymmetry can be due to many reasons other than publication bias. We also do not know with any certainty that publication bias is the true cause of funnel plot asymmetry. As such, we remain cautious in interpreting the results.

## Discussion

In this systematic review and meta-analysis, we describe summary estimates of the prevalence of hypertension and its trend in Bangladesh. Our results present a comprehensive view of the burden of hypertension in Bangladesh. Our findings suggest that the pooled prevalence of hypertension varied widely between 1.10% and 75.0% and an overall pooled prevalence of hypertension was estimated to be 20%. Prevalence was observed higher in females, in urban areas, and in the “≥ 60 years” age group. Overall, an increasing trend of hypertension was also observed.

Our findings of the overall pooled prevalence of hypertension (20%) in Bangladesh are quite similar to the global prevalence of hypertension, which is observed 22% in 2015 among adults aged 18 years and older [71]. The prevalence of hypertension among adults varies across the globe with highest prevalence observed in African region (27%) while lowest observed in American region (18%) [72]. A declining trend in hypertension prevalence was observed in high-income countries while stable or increasing trend in prevalence was observed in low- and middle-income countries [71, 72]. According to the World Health Organization (WHO) report published in 2018, prevalence of hypertension (defined as SBP and/or DBP ≥ 140/90 mmHg) among adults (aged 18 + years) in Bangladesh is 21% which is not different from what we observed in our findings [72]. While comparing our findings with reports from other neighboring regions, we also see similar results. The WHO reported a prevalence of hypertension among adults in neighboring India, Pakistan, Nepal, and Sri Lanka as 24%, 25%, 26%, and 24% respectively in 2015 [72]. Like Bangladesh, an increasing trend in the prevalence of hypertension was also observed in these low- and middle-income countries [72]. Hypertension in general is less prevalent in women, compared with men, particularly among the young. However, it is more prevalent among the elderly women than men [73–75]. In addition, the difference in hypertension prevalence between men and women varies across the global regions. For example, prevalence of hypertension was reported higher among women than men in Arab countries [76]. In neighboring South Asian countries, there is not much difference in hypertension prevalence between men and women [72]. Our contrary findings of higher hypertension prevalence in women could be explained by the higher obesity and less education in women, and potentially older women participation in the study, as all of them are important risk factors for hypertension prevalence. Further, social traditions (e.g., women should bear children and look after their family, which limit women’s physical activities) can also play a vital role in increased prevalence of hypertension among women in Bangladesh.

The factors behind the increased prevalence of hypertension in the Bangladeshi adult population could be multifarious [13]. In addition to the common risk factors for hypertension such as a high salt intake, being obese, the excessive use of alcohol, physical inactivity, stress, air pollution and smoking [77], other factors including a regional change in disease patterns from communicable to non-communicable diseases, rapid urbanization, and obsession for embracing western lifestyle could also influence high hypertension prevalence [13, 14, 78, 79]. Fast-Growing unplanned urbanization as seen in developing countries can potentially lead to change in lifestyles (e.g., change in physical activity, diet, environment, and stress) [13]. In developing countries like Bangladesh, lifestyle, environment, and dietary patterns are quite different in urban and rural areas [14]. These factors are closely linked with a higher prevalence of hypertension and are evident in our study where a higher pooled prevalence of hypertension observed among the urban population (25% compared to 15% in the rural population). An extremely high between-study heterogeneity in the prevalence of hypertension was observed. Consequently, we carried out subgroup analysis according to the cut-off level used to define hypertension, types of prevalence, gender, age group and geographical location of the study participants. However, heterogeneity was still observed within all the subgroups and within all the groups of subgroups and we could not explore the possible source of heterogeneity. We observed evidence of small study effect (p < 0.001), in which smaller studies reported a higher prevalence of hypertension. Publication bias was also indicated by the asymmetry of the funnel plot.

The strength of this study is the comprehensiveness of the process, which is a search of three different databases, well-defined inclusion/exclusion criteria, and extensive use of reference lists. Consequently, there is little chance that any relevant studies would have been missed. However, there are also several limitations to our study. We could not consider non- English publications and local journals that are not available through major international databases. In addition, a lack of uniform definition of hypertension, and the large variations in the age of the study participants in the included studies can potentially limit the comparability of our findings. As such, pooled prevalence estimates derived from the subgroup analysis can be more accurate and reliable in presenting hypertension prevalence than the overall pooled results. We identified few studies, which potentially considered adolescents with adults as study participants. In those studies, only the lower limit of the study participants’ age was provided without any information on the prevalence of hypertension among adolescents. Considering the difference in definition of hypertension for the adults and adolescents, excluding them from the analysis could potentially strengthen our study findings. The differences in the research design of the studies may also influence pooled estimates and can be a potential source of heterogeneity, which we could not explore. Prevalence estimates of hypertension are anticipated to be more precise if studies reported adjusted (for age and gender) estimates. However, most of the studies reported unadjusted prevalence and only a few studies reported age and gender-adjusted hypertension prevalence a point to be noted. Although we identified the existence of high heterogeneity in the studies and attempted subgroup analysis to overcome the issue, heterogeneity still remains and failing to explore potential sources of heterogeneity is another limitation of our study. Performing further subgroup analyses within subgroups or meta-regression according to study setting, sample size, publication year and so on may help to explore the sources of heterogeneity. Nevertheless, despite the high heterogeneity of the studies included in our review, the derived pooled estimates are reliable and within the range of prevalence of hypertension described in neighboring countries [72]. In our systematic review, we did not find rigorous population based cross-sectional study with large sample size conducted in the Bangladeshi population for estimating hypertension prevalence. Despite our meta-analysis approach, the lack of comprehensive studies in Bangladeshi population needs to be noted. We also suggest being cautious while interpreting our study findings. Bearing in mind prevalence of hypertension can vary due to the cut-off levels used to define hypertension, age groups, gender and geographical location of the study participants, the interpretation of findings should be made accordingly.

## Conclusions

In this review, we tried to systematically evaluate the scientific literature available on the prevalence of hypertension in Bangladesh and attempted to provide comprehensive summary estimates of hypertension prevalence along with its trend. Although, hypertension prevalence varies according to the criterion used to define hypertension, gender, age group and geographic area of subjects studied, the prevalence of hypertension is still high and rising in Bangladesh according to our review. Similar to WHO’s target for a 25% relative reduction in hypertension prevalence by 2025 [80], programs targeted towards primary prevention needs to be initiated to mitigate a further increase in the prevalence. Modifiable risk factors for hypertension, particularly those related with lifestyles need to be identified and addressed. Increased public awareness and knowledge on high hypertension prevalence, its risk factors, its consequences and its burden along with education programs on diet and healthier lifestyles can be helpful to prevent and confront the issue. Further, well-designed, nationwide, population-based surveillance on hypertension is warranted to provide more up to date, precise and representative estimates of its prevalence.

## Data Availability

The full list of data and the data entries for all included studies is provided in the paper. No additional supporting data is available.
